# Automated noninvasive epithelial cell counting in phase contrast microscopy images with automated parameter selection

**DOI:** 10.1111/jmi.12726

**Published:** 2018-07-12

**Authors:** R. FLIGHT, G. LANDINI, I.B. STYLES, R.M. SHELTON, M.R. MILWARD, P.R. COOPER

**Affiliations:** ^1^ Physical Sciences of Imaging in the Biomedical Sciences Doctoral Training Centre University of Birmingham, Edgbaston Birmingham B5 7EG U.K.; ^2^ School of Dentistry University of Birmingham, Edgbaston Birmingham B5 7EG U.K.; ^3^ Department of Computer Science University of Birmingham, Edgbaston Birmingham B12 2TT U.K.

**Keywords:** Cell cultures, growth curve, phase contrast microscopy

## Abstract

Cell counting is commonly used to determine proliferation rates in cell cultures and for adherent cells it is often a ‘destructive’ process requiring disruption of the cell monolayer resulting in the inability to follow cell growth longitudinally. This process is time consuming and utilises significant resource. In this study a relatively inexpensive, rapid and widely applicable phase contrast microscopy‐based technique has been developed that emulates the contrast changes taking place when bright field microscope images of epithelial cell cultures are defocused. Processing of the resulting images produces an image that can be segmented using a global threshold; the number of cells is then deduced from the number of segmented regions and these cell counts can be used to generate growth curves. The parameters of this method were tuned using the discrete mereotopological relations between ground truth and processed images. Cell count accuracy was improved using linear discriminant analysis to identify spurious noise regions for removal.

The proposed cell counting technique was validated by comparing the results with a manual count of cells in images, and subsequently applied to generate growth curves for oral keratinocyte cultures supplemented with a range of concentrations of foetal calf serum. The approach developed has broad applicability and utility for researchers with standard laboratory imaging equipment.

## Lay description

The ability to determine the number and growth rates of cells in *in vitro* cultures of cells has many useful applications, for example in toxicology and drug discovery. Commonly applied methods currently used for counting cells have several disadvantages, including destruction of the cell cultures, large user error and time‐consuming procedures. Phase contrast (PC) microscopy is a widely available type of microscopy that generates contrast in transparent cell cultures without the need for fixation and staining, and allows noninvasive imaging. However, PC image artefacts make it difficult to identify cells easily by means of computational image analysis. This paper describes a method to overcome these artefacts to enable segmentation and counting of cells from PC images. A spatial logic called discrete mereotopology was used to incorporate information regarding the image composition in terms of the tentatively identified cells to find the optimal imaging parameters and maximise the accuracy by removing incorrectly segmented regions. The results obtained overcame many of the limitations associated with standard laboratory cell counting methods.

## Introduction

Epithelial cells typically provide a barrier or lining function and can form stratified structures, for example in skin and masticatory mucosa, where a robust response to mechanical stress and chemical irritants is essential to maintaining health. Keratinocytes cultured *in vitro*, form two‐dimensional (2D) monolayers, which are frequently used to study cell behaviour; their proliferation and differentiation in response to stimuli is commonly assessed using cell counts at multiple time‐points. Determining cell numbers in cultures is laborious and traditionally involves dissociating cells from a substrate, resuspending in a known volume and manually counting the cells using a haemocytometer viewed with a microscope. Because that technique requires destruction of the cell culture, multiple measurements on the same culture cannot be made. Along with operator error, this therefore requires several replica cultures to generate growth curves (Biggs & Macmillan, 1948) consuming significant time and resource. Automated procedures to enable relatively rapid cell counting from light microscopy images of cell suspensions have been previously proposed (e.g. Logos Biosystems, 2018). However, whilst operator variability is reduced, the destructive nature of the approach remains, as does the relative expense. Furthermore, commercially available image analysis software used to count cells is often proprietary, thus algorithms are not openly published. Consequently it is not possible to determine whether anomalous results are valid or represent shortcomings of the algorithms used, or to compare studies performed using different methods.

Indirect spectrophotometric and fluorometric methods to determine cell numbers have also been developed. For example, the 3‐(4,5‐dimethylthiazol‐2‐yl)‐2,5‐diphenyletetrazolium bromide (MTT) assay is a relatively simple and inexpensive assay that can be applied to large sample numbers (several other tetrazolium compounds are now commercially available which operate similarly) (Riss *et al*., 2016). The assay involves the addition of the MTT substrate to cell cultures, where the enzymatic activity in viable cells reduces MTT to formazan (a purple water‐insoluble dye) and its concentration is then determined spectrophotometrically (Mosmann, 1983). Cell numbers are then estimated indirectly using a calibration curve. This approach is also destructive to cell cultures, error prone and requires the establishment of multiple cultures for longitudinal studies.

Fluorescence microscopy is frequently utilised to visualise otherwise transparent cells and allow high‐contrast images to be obtained for computational analysis. However, fluorescent staining techniques have a number of limitations that make their use impractical for monitoring cultures over several days to generate a growth curve. For example, nuclear stains such as DAPI or Hoechst intercalate DNA and are excited by wavelengths in the UV wavelength range of the electromagnetic spectrum, thus samples must be irradiated with UV light. Both of these visualisation features have the potential to cause mutagenic effects thus neither dye is suited to the application of monitoring cells over relatively long incubation time‐periods. Furthermore, DAPI in particular has low permeability in live cells, thus cultures are usually fixed in order to obtain useful levels of intensity, thereby precluding longitudinal use. A further approach for introducing fluorophores into live cells is to use gene transfer techniques, which can enable cells to stably express fluorescent proteins, for example green/red fluorescent protein. This approach has the advantage of generating a heritable fluorophore; however, transfection is rarely undertaken for the purpose of cell enumeration because cells may not be transfected with equal efficiency. Furthermore stable transfection is difficult to achieve consistently in some cell lines (Karra & Dahm, 2010).

Bright field light microscopy, in principle, lacks contrast in focused images thus it is unsuitable for analysis of unstained cells. However, defocusing the image can increase contrast due to shifts in the light path lengths causing constructive or destructive interference, although this comes at the expense of the image resolution. Dehlinger *et al*. (2013) showed that when the imaging plane was moved marginally above the focal plane, contrast increased such that cell centres became brighter than the background, and that the opposite effect was observed when the imaging plane was moved below the focal plane, that is cell centres became darker. Dehlinger and coworkers used that principle to locate cells in a monolayer by subtraction of two such bright field microscopy images acquired with the objective lens displaced by 15 μm (Dehlinger *et al*., 2013). However, this relatively small distance is difficult to set consistently without motor‐controlled focus adjusters.

**Figure 1:**
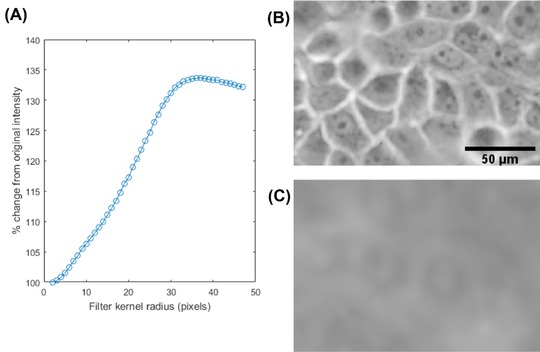
*Contrast change after mean filtering PC images*. (A) Graph showing the average change in intensity in sample points inside cells after mean filtering with a range of kernel radii. Note how intensity increased to a maximum at r=34. (B) In‐focus PC image of H400 cells. (C) Image A mean filtered with kernel radius, r=34 pixels (the radius at which the maximum is observed in (A). Intensity became higher inside cell regions, although image resolution was lower than the focused image. The scale bar for all images is shown in (B).

Phase contrast (PC) microscopy uses the principle of translating small changes in the phase of transmitted light into changes of light amplitude to enhance contrast in optically transparent objects without the requirement for defocusing. However, there are unavoidable artefacts in PC in the form of a ‘halo effect’: regions of high light intensity at the edges of objects (Fig. 1A) that limit differentiation of specimens from the background on the basis of intensity alone. To work around these artefacts, PC imaging techniques fall mainly into two categories: textural analysis to distinguish cell‐populated regions from background regions, and high‐precision segmentation of individual cells. Methods in the first group are unable to provide information relating to individual cells and are therefore not suitable for use at very low cell densities (Sommer *et al*., 2011; Jaccard *et al*., 2014). Conversely, methods in the second group are unsuitable for cell segmentation in images with high cell density which require time‐consuming initialisation and high computational power for analysis (Seroussi *et al*., 2012). Neither of these approaches is appropriate for both low‐ and high‐density images of cell cultures, as is required to generate comprehensive growth curves.

An alternative approach to locating cells in PC images involves correcting for the halo artefact using mathematical models of PC optics to deconvolve images (Yin *et al*., 2012). However, this approach is computationally expensive when used on large images. Additionally, *a priori* parameters required for deconvolution, such as the diameter of the microscope phase ring, are not consistently provided by microscope manufacturers. A more rapid, approximated form of deconvolution has been used to locate epithelial cells in ‘scratch wound’ assays using a ’difference of Gaussians’ filter but to our knowledge this has not been used for cell counting (Sarsby *et al*., 2012). Likewise, Laplacian of Gaussian filter has been used to detect cells in PC images (Smith *et al*., 2008). However, careful selection of radius and standard deviation parameters is essential in order to optimise detection rate in such filters (Yin *et al*., 2012). Scale‐invariant feature transform (SIFT) techniques offer robust performance, but as patented technique it is not freely available to deploy or implement as part of other software (Lindeberg, 1994; Lowe, 2004).

Other live cell imaging algorithms have been developed which enable cell tracking from videos acquired by PC microscopy (Li *et al*., 2008). However, these may not be useful for the purpose of counting cells for generation of growth curves because: (1) The microscope may not be used for any other purpose during the time of the experiment. (If cell growth is to be monitored over the course of multiple days, this drastically limits the data collection in other concurrent experiments – this is problematic in busy laboratories.) (2) Only one culture may be imaged at a time so replicate cultures cannot be evaluated concurrently. (3) There is a need of stage incubator with a CO_2_ supply to maintain culture conditions for long periods of time, and this is also costly to maintain.

Because the effect of defocusing causes the image to lose contrast detail (similarly to digital blurring), we investigated whether convolution filtering could be used on PC images to emulate the contrast changes in the defocused bright field microscopy and enable segmentation of regions corresponding with cells to estimate numbers. The aim of this work was therefore to develop a nondestructive, image analysis‐based method based on this concept, for achieving accurate cell enumeration for the purpose of generating growth curves over a period of several days. To increase utility, applicability and productivity, the method was designed without the requirement for costly automated equipment such as microscope incubators or motorised stages. An additional aim of this work was to overcome the issue of parameter selection suffered by other techniques through development of a novel automated method for parameter optimisation.

The remainder of this paper is organised as follows: first the principle is outlined, including parameter selection, noise removal and details of how to apply the method to generate growth curves. Subsequently, materials and methods are described and the results obtained using the novel method are validated using a comparison with manual counts of cells in images at a range of densities and application to an alternative epithelial cell line. An application of the method is demonstrated for growth curve generation in oral keratinocyte cultures supplemented with a range of foetal calf serum (FCS) concentrations that influences cell growth rates.

## Computational method development

### Principle of cell localisation

The principle of defocusing microscopy suggested by Dehlinger *et al*. (2013) consists of varying the image plane above and below the focal plane to generate two images, where cells have darker and brighter centres respectively, and then subtracting these to generate a new image that can be relatively easily segmented (e.g. using a traditional intensity thresholding technique). In focused PC microscopy images generally cell centres appear darker than cell edges without the requirement for defocusing (Fig. 1B). To generate a second image in which cell centres appear brighter than cell edges, a mean filter was considered for two reasons. First, because it provides an equal contribution of all pixels in the filter kernel to the final filtered value, this means that when the kernel is centred on a pixel at the centre of a cell and it is large enough to encompass the bright edges, the average intensity of the cell centre will be brighter. Second, unlike a Gaussian or Laplacian filters, which require parameterisation of both a kernel radius and a standard deviation, a mean filter is described only by a single radius value. Therefore, through careful selection of the kernel radius, a mean filter has the potential to mimic the bright cell centres observed in defocused images with minimal parameterisation required.

This concept was investigated in a set of images where various mean filters with kernel radii, 2<r<47 pixels were applied to in‐focus PC images of H400 oral keratinocyte cells (henceforth referred to as H400 cells) using a ×10 objective. The intensity of ten randomly sampled pixels located in cell cytoplasmic regions was measured after applying each filter size to estimate the average cell cytoplasm intensity. It was found that the average intensity in the cell centre increased up to a maximum at *r* = 34 pixels (Fig. 1A).

**Figure 2:**
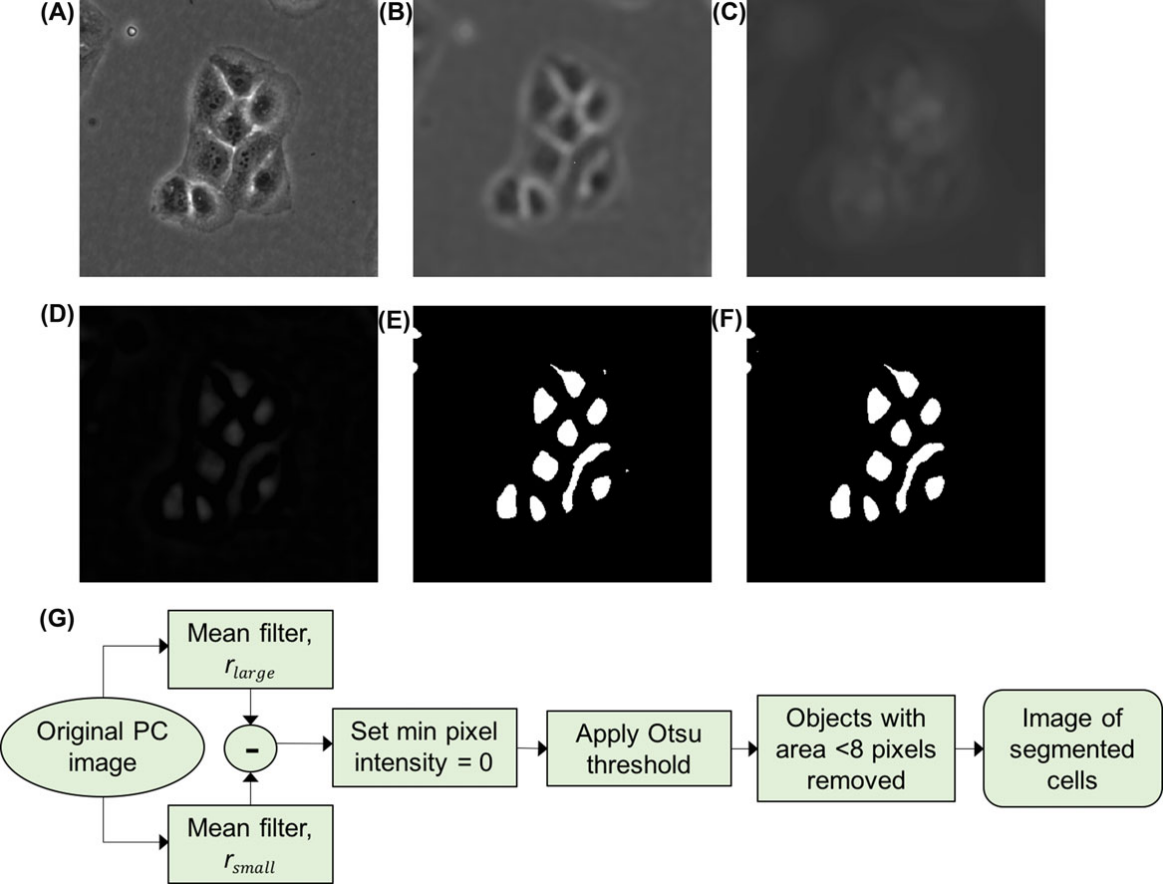
Proposed workflow (G) for segmentation of cells in PC microscope image (A). Mean filters with radii $r_{small}$ and $r_{large}$ were applied (images B and C respectively) and these were subtracted from each other (D) before application of the Otsu threshold to binarise the image (E). Very small objects with area of less than 8 pixels were removed and the cell number was determined by the number of binary objects in the final segmented image (F).

Subsequently we investigated whether cells could be segmented for counting through subtraction of two versions of the same PC image filtered with different sized mean filters such that $r_{small}$ smoothed fine detail inside cells with minimal change in intensity (Fig. 2B) whereas $r_{large}$ resulted in intensities inside cells increasing to their brightest point (Fig. 2C). An intensity‐based threshold could then be applied to the image resulting from subtraction (Fig. 2D) to produce an image of binary regions representing cells. The proposed algorithm is shown as a workflow in Figure 2(G). A minimum area condition was implemented to remove small (noise) regions with an area of less than 8 pixels (9 μm) (chosen empirically as these were unlikely to represent a cell), and the number of remaining binary regions taken as the number of cells.

### Parameter selection

To optimise the number of cells represented correctly in the final image by a single binary region, a robust parameterisation for determining $r_{small}$ and $r_{large}$ was required, because the filter radii can affect the rate of occurrence of mis‐segmentation events and consequently the accuracy of the cell counts. Examples of mis‐segmentation events include ‘merged’ and ‘split’ cell regions caused by over‐ or undersmoothing by $r_{small}$, and undetected cells or ‘missed’ events, which can occur when $r_{large}$ is not optimised. Spurious ‘noise’ regions not associated with cells also contribute to errors in the cell count.

**Figure 3:**
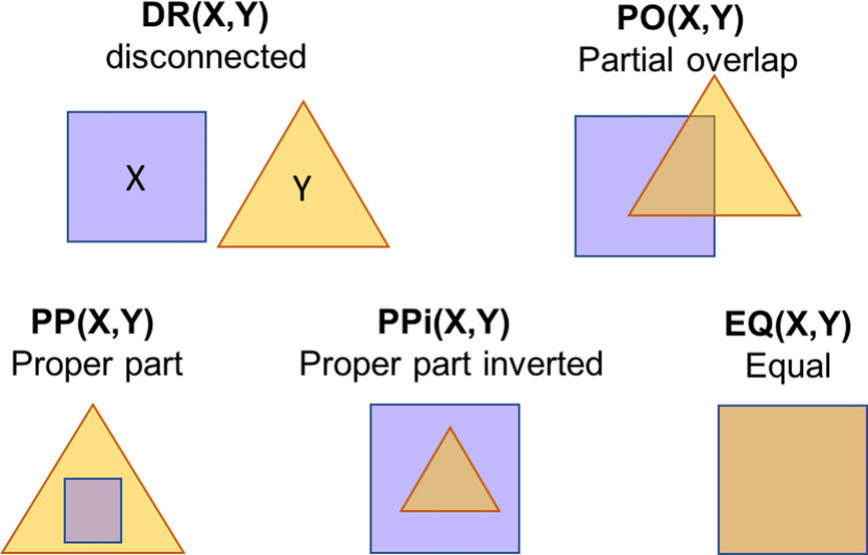
The RCCD5 relationship set. The five possible parthood relations between binary objects X and Y are defined by the RCCD5 set. The abbreviations indicate: DR(X,Y) – ‘X and Y have a disconnected relation’, PO(X,Y) – ‘X partially overlaps with Y’, PP(X,Y) – ‘X is a proper part of Y’, PPi(X,Y) – ‘X is a proper part inverted of Y’ and EQ(X,Y) – ‘X is equal to Y’ (Randell *et al*., 2013).

To investigate the degree of matching between the segmentation results and ground truth cell positions, we used discrete mereotopology (DM), a spatial logic which describes the possible relationships between binary regions pairs in discrete space (Galton, 1999). DM was used to incorporate biological structure information programmatically to assess the success of different combinations of filter kernel radii for cell detection, while taking as a ground truth nuclei detected in epifluorescent images of the same culture stained with Hoechst dye (see the Materials and Methods section) obtained concurrently with PC images. Note that Hoescht dye is not suitable for longitudinal imaging due to cell toxicity and was used here solely for the purpose of generating a ground truth data set for parameterisation. The region connection calculus relation set RCC5D is an implementation of DM which describes a set of five possible relationships between two regions in discrete 2D space (Fig. 3) (Randell *et al*., 1992).

**Figure 4:**
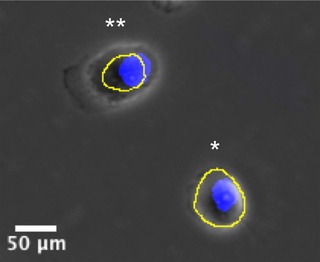
Examples of acceptable cell detections. PC images of H400 cells with manual example cell segmentations shown in yellow and nuclei stained with Hoechst in blue. The cell indicated by * indicates the expected case for a correct detection, PP(N,C), the detected nucleus N is a proper part of the cell segmentation C (green outline). Due to cell undersegmentation, the condition is relaxed to include a partial overlap relation, PO(N,C), such as the cell indicated by **.

The filter parameter selection was performed as follows: initially, the desired relationship between the segmented and the detected nuclei was defined in terms of RCC5D relations. H400 cells generally have a single nucleus (N), entirely contained within the cell body or for simplicity, the cytoplasm (C). In the ideal case, the segmented region should correspond with the cell body, thus the following constraints defined a correctly detected cell:

- A nucleus is a proper part of (i.e. PP(N,C)) or equal to (i.e. EQ(N,C)) the segmented cell region. Although PP is the ideal expected (Fig. 4), the restriction was relaxed to also include partial overlap (PO(N,C)) and equal (EQ(N,C)) relations to account for cases when the cell is a slightly under‐segmented region but still detected (PO(N,C) case (Fig. 4) or when the cytoplasm is minimal, not detected at the imaging resolution and N and C appear to coincide (EQ(N,C) as often seen in cells such as lymphocytes in histological preparations)
- There should be an exclusive one‐to‐one relationship between pairs of segmented cell regions and nuclei (i.e. our definition does not include multinucleated cells).

**Figure 5:**
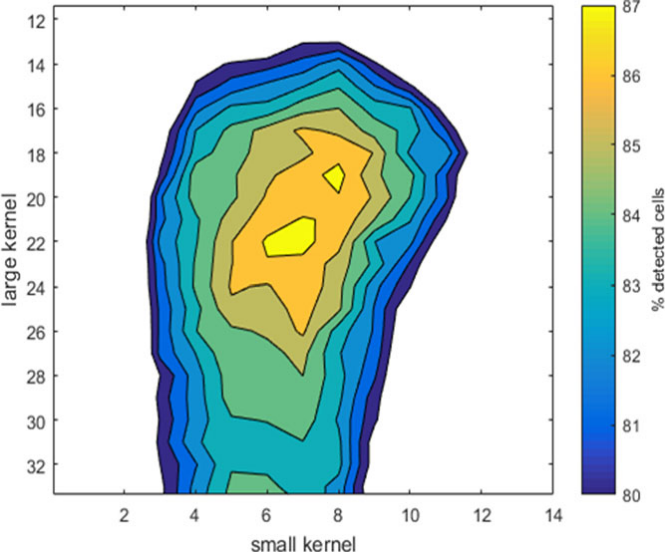
Contour map showing the percentage of 1354 H400 cells correctly detected in phase contrast images, for a range of parameter combinations.

**Figure 6:**
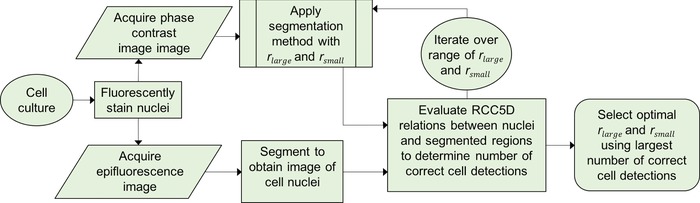
Workflow for selection of parameters using stained nuclei as ground truth. The segmentation method workflow is given in Figure 2.

The dataset used here consisted of 1354 H400 cells in four images of varying sizes at a range of densities stained using Hoechst dye. Finally, we performed an exhaustive search over a range of $r_{small}$ and $r_{large}$ filter kernel radii to segment the PC images and assess the rate of correct cell detections achieved. The number of correct detections achieved by the various parameter pairs for the H400 cell dataset is shown as a contour map in Figure 5. The parameter pair with the highest percentage of correct detections (87.1%) was $r_{small}=\;$ 7 pixels (6 μm) and $r_{large}=\;$ 22 pixels (20 μm). These values were considered the optimal parameters, which were applied subsequently to segment all images analysed. The workflow for parameter selection using the nuclear ground truth method is shown in Figure 6.

### Noise removal

**Figure 7:**
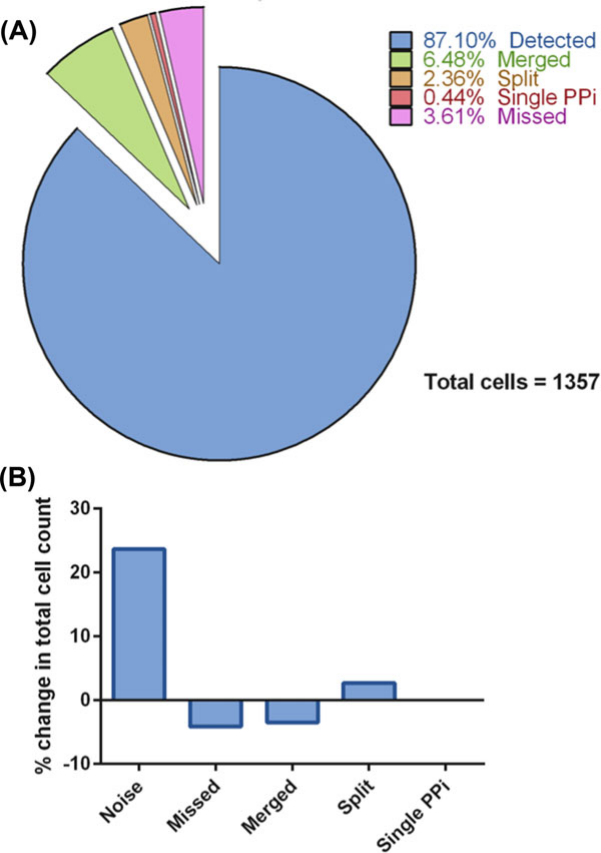
Erroneous misdetection rates for H400 cells segmented using $r_{small}$  = 7 pixels and $r_{large}$ = 22 pixels. (A) Pie chart showing how each cell in the parameter training set is labelled using the final parameters of $r_{small}$ = 7 pixels and $r_{large}$ = 22 pixels. (B) Bar chart showing the types of error leading to count error and their contribution to the change to the true count. Noise segmentations were the biggest contributor to errors in the cell count.

Errors in the final cell count were quantified using the ground truth dataset to label each segmented region in the final result as correctly detected or as misdetected according to how the definition of a ‘correct detection’ is violated. The percentage contribution to the error in total cell numbers for each misdetection type is shown in Figure 7. Noise regions exerted the greatest effect, increasing the cell count by 24%, thus this type of error was the focus for subsequent correction.

Noise removal aimed to maximise the detection and removal of artefactual regions whilst minimising the misclassification of correct cell detections. Linear discriminant analysis (LDA) was used for this purpose. The ground truth images of cell nuclei were used to label the binary objects in the final segmented image according to satisfaction or violation of the DM constraints for correct detection and served as a labelled training set. The dataset contained 1175 cell regions, 41 merged regions, 70 split regions and 280 noise regions. A very small fraction of detections were PPi(N,C). The PPi relations indicate erroneous segmentations that should not have been included in the definition of cell detection for parameterisation, but which are not necessarily counting errors because they still meet the exclusive one‐to‐one relationship condition between a nucleus and a segmented region. PPi events accounted for only 0.44% of cell segmentations and were not considered further as a candidates for removal. Merged and split objects were relabelled as cells because the morphological and greyscale properties of such objects were more similar to cells than noise. Such objects had similar but opposite effects on the total cell count and in effect cancelled each other out. The features used for classification were morphological properties of the binary segmented regions and greyscale properties (shown in the Supplementary Information) using the Particles8 plugin for ImageJ (Landini, 2018).

LDA gave an overall rate of correct classification of 96.4%, retaining 96.3% of cell regions whilst removing 78.9% of noise. The F1 score was 0.959 (as calculated using Eq. (4)). The discriminants trained on this dataset were used to remove noise on all further H400 cell images analysed using the same apparatus.

### Determining the total number of cells in whole cultures from PC images

H400 cells grow in discrete colonies from single cells that adhere to the substrate, to form confluent monolayers over time. Prior to confluence this results in variations in cell numbers detected at different locations of cultures. To account for such variation in cell density, the mean number of cells in multiple images acquired at random locations, ${\bar{N}}_{image}$, was used to estimate the total number of cells in the culture, using the ratio of substrate area and image area. For example, in the case of an image of a culture in a 35 mm dish the calculation used was as follows:

(1) $$\begin{array}{ccc} \;N_{\mathrm{total}} & = & \frac{A_{\mathrm{total}}}{A_{\mathrm{image}}}\;\times \;{\bar{N}}_{\mathrm{image}}=\frac{962.11}{1.06}\;\;{\bar{N}}_{\mathrm{image}}=\;907.65\\ & & \times {\bar{N}}_{\mathrm{image}}. \end{array}$$

**Figure 8:**
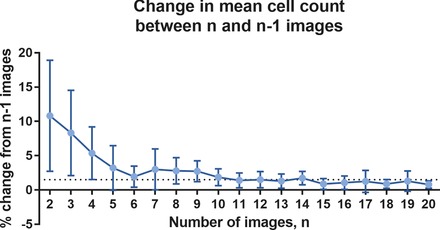
The magnitude of the change in mean cell count per image, ΔC when the number of images included in the mean count calculation was increased from n−1 to *n*. The dotted line indicates ΔC=1.5. ΔC was calculated as the average of 18 image sets and the error bars indicate standard deviation.

As the area included in the image set tended towards the total area of the culture vessel, the accuracy of the estimated $N_{total}$ should approximate the true count value. However, increasing the number of images acquired requires cell cultures to be outside the desired incubation conditions (temperature and atmosphere) for long periods of time and that also increases the risk of contamination. To investigate how the mean cell number per image changed as the number of images *n* increased, sets of 20 images were acquired at random locations in H400 cell cultures in triplicate 35 mm culture dishes. Eighteen image sets were acquired at multiple time points to include cultures at different levels of cell density. The magnitude of the difference between the mean cell count calculated using *n* images and n−1 images, ΔC, was averaged over the 18 image sets and plotted against *n* (Fig. 8). The graph shows an initial rapid reduction in ΔC with the number of images included in the calculation of the mean and a stabilisation to approximately 1.5% at n=11. After this point there were minimal improvements in ΔC, thus 11 was selected as the number of images acquired and analysed in subsequent procedures.

## Materials and methods

### Cell culture

H400 cells (an epithelial cell culture derived from a human oral squamous carcinoma) are an adhesive cell line which have been used as an *in vitro* model of the oral mucosa (Prime *et al*., 1990). Cells were maintained at 37°C in a humidified atmosphere with 5% CO_2_ in Dulbecco's MEM/nutrient mix (Sigma, UK) supplemented with 10% foetal calf serum (FCS) (Biosera, UK), 0.6 μg mL^–1^ L‐glutamine (Sigma, UK) and 0.4 mg mL^–1^ hydrocortisone (Sigma, UK).

For validation and experimental analysis H400 cells were initially seeded into 35 mm culture dishes (Sarstedt, UK). To reduce the likelihood of nonuniform cell adhesion across the vessel due to uneven temperature distributions, culture dishes were preheated to 37°C prior to seeding and briefly gently agitated following seeding to ensure conformity of cell mixing.

For experiments that generated growth curves to investigate the dose‐dependence of H400 cells on FCS concentration, duplicate cultures were seeded as described above at an initial concentration of 5 × 10^4^ cells and supplemented with 10, 5 and 2.5% FCS, respectively, in a total volume of 2 mL of media.

The U2OS human bone epithelial osteosarcoma cell line was derived from bone tissue of a 15‐year‐old female patient (Ponten & Saksela, 1967). U2OS cells are commonly used to model osteoblast behaviour *in vitro* (Rehman *et al*., 2013). U2OS cells were maintained in McCoy's 5A Modified Medium with L‐glutamine and sodium bicarbonate supplemented with 10 mL L^–1^ penicillin–streptomycin and 10% FCS.

### Fluorescent staining

As per the manufacturer's instructions, one drop of NucBlue® Live ReadyProbes® Reagent Hoechst stain (Thermo Fisher Scientific, UK) per millilitre of media was added to live cell cultures, which were incubated under darkened conditions for 15 min at 37°C prior to image capture as described below.

### Image acquisition and processing

Images of cells were acquired concurrently with PC and fluorescence microscopy using a Nikon TE300 microscope with a ×10 objective and a Retiga‐2000R CCD camera (Qimaging, UK) using Micro‐Manager software (Edelstein *et al*., 2014) for ImageJ (Rasband, 1997). Images (1600 × 1200 pixels) were acquired and calibrated using a stage micrometre (image size was of 1.19 × 0.89 mm). All images were saved to 8‐bit greyscale TIFF format prior to any further processing. Cultureware lids were removed before image capture to reduce image contrast degradation due to condensation. Images were acquired inside a microscope enclosure chamber cleaned with 70% ethanol to minimise the risk of contamination.

Epifluorescence images of cell nuclei for the ground truth were segmented before use in parameter selection. This was done using ImageJ v1.51m (Rasband, 1997). This was undertaken by first smoothing using a mean filter with a 3 × 3 kernel and normalising the intensity histogram to the full range of 8‐bit image values. The mean local threshold method was applied with a kernel radius of 20 pixels (Gonzalez & Woods, 2007). A watershed function was applied to separate densely packed nuclei and the resultant binary objects were eroded twice to remove small protrusions and ensure nuclear regions were not over‐segmented. Because correct nuclear segmentations were of utmost importance for correct parameter selection, images were inspected visually and manually corrected if necessary using the ImageJ paint tool. This empirical method was suitable for proof of concept but can be optimised further using automated methods.

Analysis of DM relations between nuclear and PC segmentations for the purpose of parameter selection utilised ImageJ RCCD plugins (Landini *et al*., 2013; Randell *et al*., 2013). Greyscale and morphological features of PC segmentations used to classify binary regions for the purpose of noise removal were obtained using the Particles8 plugin for ImageJ (Landini, 2018). Plugins for ImageJ used in this work are freely available at: http://www.mecourse.com/landinig/software/software.html. Graphs representing correct detections for parameter combinations and LDA were generated using MATLAB version 2015b and the MATLAB Classification Learner App.

Manual counting of the number of cells present in images was achieved by an experienced microscopist using the ImageJ CellCounter plugin to click on each cell (Rasband, 1997). Images containing cultures with a range of cell densities were used and the counter was blinded to the data generated by the image analysis technique.

### Classification measures

The success of classification of objects into a class, *X* was measured using a number of metrics calculated using four possible classification outcomes (Powers, 2011). These were as follows:

- true positive (TP) – the classifier correctly identifies an object as *X*
- true negative (TN) – the classifier correctly identifies an object as not *X*
- false positive (FP) – the classifier incorrectly identifies an object as *X*
- false negative (FN) – the classifier incorrectly identifies an object as not *X*

Classification precision (*p*) indicates the fraction of objects correctly classified as *X* out of the total number of objects classified as such, and was calculated as follows:

(2) $$p\;=\frac{\mathrm{TP}}{\mathrm{TP}+\mathrm{FP}}\;.$$

Classification recall (***r***), or sensitivity, indicates the fraction of objects correctly classified as ***X*** out of all true ***X*** objects, and was calculated as follows:

(3) $$r\;=\frac{\mathrm{TP}}{\mathrm{TP}+\mathrm{FN}}\;,$$

where ***r*** and ***p*** were expressed as percentages.

The *F*_1_ score is the weighted average of precision and recall and serves as a convenient single measure of classification success that takes both parameters into account. An *F*_1_ score of 0 indicates no agreement between classification labels and true values, and 1 indicates complete agreement. *F*_1_ was calculated as follows:

(4) $$\;F_{1}=\;2\left(\frac{\mathrm{pr}}{p+r}\right).$$

## Results

### Validation against manual image counts

**Figure 9:**
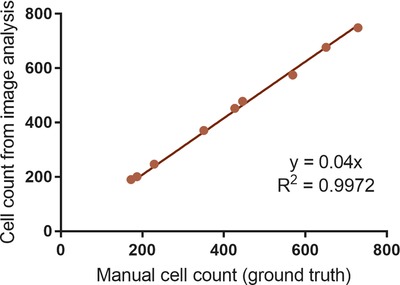
Correlation of image analysis cell counts with manual image counts. Each point corresponds to a single phase contrast microscope image of H400 cells.

The new method was validated through comparison with manual counts of cells in 9 images across a range of cell densities. A curve of the form

(5) $$\;\mathrm{Coun}t_{\mathrm{image}}=\;\mathrm{gradient}\times \mathrm{Coun}t_{\mathrm{suspension}}$$

was fitted using linear regression to examine whether the two methods were linearly correlated (Fig. 9).

The resulting gradient from comparison of the two techniques was 1.04 which indicated that the count from the image analysis method was only on average 4% higher than the manual count, and the two methods showed a very high degree of correlation (*R* ^2^ > 0.99).

### Application to U2OS cell line

U2OS cells were selected for experimentation because, like H400 cells, this cell line displays regular epithelial morphology and forms nonoverlapping monolayers when cultured *in vitro* and are often used as a model for bone tissue (Rehman *et al*., 2013; Zhao *et al*., 2015).

When parameterisation was performed, a peak correct cell detection rate of 92% was found for parameter values $r_{small}=\;$ 9 pixels (8 μm) and $r_{large}=\;$ 30 pixels (27 μm). LDA performed as previously provided a *F*_1_ score of 0.97, retaining 97.7% of cells and removing 78.2% of noise. These values are comparable, if not superior, to the results obtained for the H400 cell line.

### Application of the image analysis method to foetal calf serum supplemented cultures

**Figure 10:**
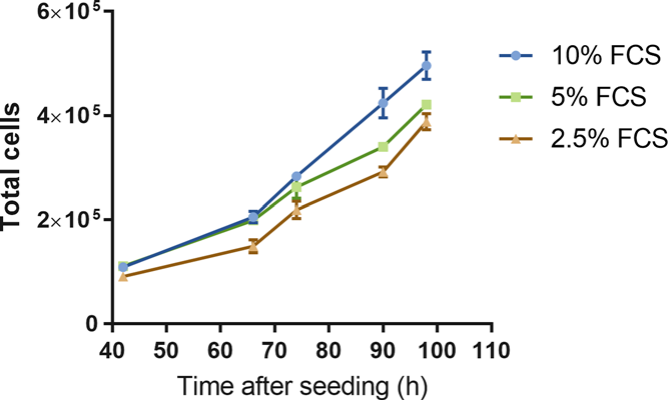
Growth curves for H400 cells supplemented with 10%, 5% and 2.5% FCS as measured using the image analysis cell counting method. The mean of duplicate plates is plotted at multiple time points and error bars show standard deviations.

*In vitro* eukaryotic cell cultures are commonly supplemented with FCS to provide a source of growth factors and nutrients required for survival and proliferation. Cultures supplemented with lower levels of FCS generally exhibit lower rates of cell growth (Oya *et al*., 2003). Duplicate cultures of H400 cells supplemented with the standard FCS level of 10% as well as reduced levels of 5% and 2.5% were used to validate the cell counting method and demonstrate that it was able to differentiate different cell growth rates. Cultures were imaged at multiple time points between 48 and 98 h postseeding and growth curves were generated from the average cell counts at each time point. The image analysis method demonstrated the expected dose‐dependent growth rate of H400 cells using the different FCS supplementation dosing regime (Fig. 10).

## Discussion

This paper describes a novel method for determining cell number from PC microscopy images of adherent epithelial cell cultures. The application of discrete mereotopology overcomes the issue of subjective parameter selection faced by many image analysis based techniques because it enables image analysis parameters to be programmatically validated and it serves additionally to generate a labelled training set on which to train classifiers for noise removal. However, because image analysis parameters are specific to a given cell type and experimental setup, parameterisation and training of noise removal classifiers would be necessary at the onset of each future experiment using new imaging devices and a specific magnification. Comparable results obtained during application to the U2OS epithelial cell line suggest that this technique has application in other cell lines which have epithelial‐like morphology.

Validation of the method against a manual count of cells in images showed that the novel method was able to achieve excellent agreement with the manual ground truth aside from a systematic overestimation of 4%. This overestimation could be explained by the inability of the noise removal method to remove all erroneous regions, as discussed in the Computational Method Development section. However, despite this small systematic difference, the counts were strongly linearly correlated, thus the image analysis method was able to measure relative cell count changes. These data were also supported by the generation of growth curves from cultures supplemented with differing FCS concentrations.

The image analysis method offers many advantages over dilution‐based laboratory methods such as the MTT assay, counting using a haemocytometer or automated counting methods due to avoidance of user error and/or the lack of requirement of a cell suspension step. The noninvasive image analysis approach also enabled a longitudinal study of the effects of FCS supplementation on H400 cells over time from the same cultures. This method therefore provides an approach which has broad utility and, importantly, requires only standard laboratory equipment. The approach presented helps appears ideal to reduce costs both through avoiding expensive microscope equipment to be linked to given cultures, through reduced ongoing expenditure for reagents, and the time in generating replicate plates.
