# Supplementary material for: Automated noninvasive epithelial cell counting in phase contrast microscopy images with automated parameter selection
Source: J Microsc. 2018 Jul 12;271(3):345–54. doi: 10.1111/jmi.12726 (PMC6849568; doi:10.1111/jmi.12726)
Supplement: Supplementary file 1 [file JMI-271-345-s001.docx]

# Supplementary information

|  | Feature | Description |
| --- | --- | --- |
| Morphological properties | Perimeter ($P$) | Length of region boundary |
|  | Area ($A$) | Area inside perimeter |
|  | Feret diameter ($F$) | Length of the longest region |
|  | Breadth ($B$) | Length of the widest region perpendicular to Feret axis |
|  | Convex hull $(C_{hull}$) | Perimeter of the smallest convex polygon enclosing the object |
|  | Convex hull area ($C_{area}$) | Area of the convex hull |
|  | Minimum radius ($R_{min}$) | Radius of the inscribed circle centred at the centre of mass |
|  | Maximum radius ($R_{max}$) | Radius of the enclosing circle centred at the centre of mass |
|  | Minimal bounding circle radius | Radius of smallest circle enclosing the object |
|  | Aspect ratio | $F/B$ |
|  | Circularity | 4$\pi A/P^{2}$ |
|  | Roundness | $4A/\pi F^{2}$ |
|  | Area equivalent diameter | $\sqrt{4A/\pi}$ |
|  | Perimeter equivalent diameter | $P/\pi$ |
|  | Equivalent ellipse area | $\pi FB/4$ |
|  | Compactness | $\left( \sqrt{4A/\pi} \right)/F$ |
|  | Solidity | ${A/C}_{hull}$ |
|  | Concavity | $C_{area}-A$ |
|  | Convexity | $C_{hull}/P$ |
|  | Shape | $P^{2}/A$ |
|  | R factor | $C_{hull}/\pi F$ |
|  | Modification ratio | $2R_{min}/F$ |
|  | Sphericity | $R_{min}/R_{max}$ |
|  | Area of bounding box ($A_{box}$) | $FB$ |
|  | Rectangularity | $A/A_{box}$ |
| Greyscale properties of corresponding PC image | Greyscale integrated density | |
|  | Minimum greyscale value | |
|  | Maximum greyscale value | |
|  | Modal greyscale value | |
|  | Median greyscale value | |
|  | Average greyscale value | |
|  | Greyscale average deviation | |
|  | Greyscale standard deviation | |
|  | Greyscale histogram skewness | |
|  | Greyscale histogram kurtosis | |
|  | Greyscale entropy | |

Table 0.1 – Features calculated for segmented objects for use in noise removal techniques using the ImageJ Particles8 plugin.
